# Autonomy Support and Achievement: Exploring the Mediating Roles of Homework Mindsets and Effort

**DOI:** 10.3390/bs16020181

**Published:** 2026-01-27

**Authors:** Jianzhong Xu

**Affiliations:** Department of Counseling, Higher Education Leadership, Educational Psychology, and Foundations, Mississippi State University, Starkville, MS 39762, USA; jx18@colled.msstate.edu

**Keywords:** achievement, autonomy support, growth mindset, fixed mindset, homework effort

## Abstract

Grounded in self-determination and mindset theories, the current investigation examined whether students’ homework mindsets and effort mediated the association between autonomy support—from both teachers and parents—and mathematics grades. The study sample included 988 Chinese students in Grade 7 to 9. Structural equation modeling indicated that both teacher autonomy support and parent autonomy support had indirect positive effects on mathematics grades, with homework mindsets (fixed and growth) and homework effort serving as mediators. Additionally, teacher and parent autonomy support directly associated with homework effort, while also exerting indirect associations with homework effort via homework mindsets. Collectively, the current investigation underscores the critical roles of autonomy support, homework mindsets, and homework effort in the homework process, offering important theoretical and practical insights.

## 1. Introduction

Drawing on self-determination theory (Deci & Ryan, 2008; Ryan & Deci, 2020) and mindset theory (Dweck, 1999; Dweck & Yeager, 2019), autonomy support, mindset, effort, achievement are viewed as interrelated constructs that may be linked through direct and indirect associations (Grolnick & Slowiaczek, 1994; Feng et al., 2019; Ma et al., 2020; Zarrinabadi et al., 2021). Yet, as discussed below, prior research has not empirically examined whether mindsets and effort mediate the association between autonomy support and student achievement. This study aims to bridge this gap by examining these potential mediating mechanisms.

While the mediating roles of mindsets and effort in the link between autonomy support and achievement could be examined across a range of academic contexts, this study specifically focuses on homework for several compelling reasons. Typically referred to as “tasks assigned to students by school teachers that are meant to be carried out during nonschool hours” (Cooper, 1989, p. 7), homework has long been a globally prevalent instructional practice, deeply integrated into students’ daily academic routines (Avcı & Özgenel, 2025; Fernández-Alonso et al., 2022; J. C. Núñez et al., 2025; Xu et al., 2024). Prior research generally supports a positive association between homework and academic achievement (Cooper et al., 2006; Fan et al., 2017; Xu & Corno, 2022). As homework often lacks the structure and supervision of classroom tasks, it places greater demands on student self-direction (Katz et al., 2009; Trautwein et al., 2009; Yang et al., 2016). In such contexts, autonomy support may become even more crucial than in the more structured classroom settings (Xu, 2025).

Additionally, as “mindset theory is a theory about responses to challenges or setbacks” (Yeager & Dweck, 2020, p. 1272), homework represents a particularly relevant context in which such responses are activated. Students’ beliefs about ability—whether they perceive ability as fixed or malleable—can significantly shape the effort they devote to homework and their resulting performance. Furthermore, the homework setting offers a unique opportunity to simultaneously examine autonomy support from both teachers and parents—a combination that has received little empirical attention in relation to its joint associations with students’ mindsets (Ma et al., 2020) or effort (Feng et al., 2019) within a single empirical framework.

This area of research holds particularly significance in the context of secondary mathematics, a subject marked by a heavy homework load (Rosário et al., 2018), sustained effort demands (Marsh et al., 2016), and increasing abstraction (J. C. Núñez et al., 2015)—and globally recognized as foundational for success in STEM fields and students’ future academic and career opportunities (Maass et al., 2019). Within this challenging yet consequential context, support for autonomy from both teachers and parents may be particularly pivotal in shaping students’ mindsets and fostering effort necessary to persist with assignments—key contributors to academic success.

### 1.1. Theoretical Frameworks

#### 1.1.1. Autonomy Support and Achievement

Self-determination theory (Deci & Ryan, 2008; J. L. Núñez & León, 2019; Ryan & Deci, 2020) posits that autonomy—alongside competence and relatedness as fundamental psychological needs—plays a vital role in fostering individuals’ natural inclinations for growth, psychological integration, and well-being. In this context, autonomy is conceptualized as the experience of self-endorsement, volition, and freedom aligned with one’s personal values, goals, and interests (Ryan & Deci, 2020). Importantly, its development is strongly influenced by the social environment, particularly through the role of teachers and parents as key socializing figures. Autonomy support refers to the degree to which socializing figures—such as teachers and parents—acknowledge, understand, and respond to students’ perspectives; offer opportunities for self-directed engagement and ownership in academic tasks; and foster independent thinking and active learning participation (Ryan et al., 2016; Vansteenkiste et al., 2012). This type of support is critical, as it fosters students’ intrinsic motivation, enhances task-oriented effort, builds a sense of competence, and ultimately contributes to academic success (Ahn et al., 2021; Feng et al., 2019; Ryan & Deci, 2020).

Consistent with this view, prior research indicates that autonomy support from both teachers and parents positively influences students’ academic achievement (Ahn et al., 2021; Bindman et al., 2015; Eakman et al., 2019; Mammadov & Schroeder, 2023; Okada, 2023; Vasquez et al., 2016). For instance, in a meta-analytic review, Vasquez et al. (2016) reported that parent autonomy support was positively related to academic performance. Likewise, Okada’s (2023) meta-analysis revealed a similar positive association between teacher autonomy support and academic achievement. Together, these results underscore the need to promote autonomy-supportive environments in both school and school contexts to foster student achievement.

#### 1.1.2. The Mediating Roles of Mindsets and Effort

Individuals’ beliefs about whether their attributes—such as intellectual ability—are fixed or capable of growth significantly influence their motivation and learning (Dweck, 1999; Dweck & Yeager, 2019; Yeager & Dweck, 2020). Those who endorse an entity theory regard ability as something innate and unchangeable. Consequently, they often prioritize demonstrating their competence and often interpret setbacks—such as difficulty or failure—as evidence of limited ability, which can undermine their effort, persistence, and readiness to embrace academic challenges (Blackwell et al., 2007; Yeager & Dweck, 2020). In contrast, individuals who hold an incremental theory perceive ability as malleable and capable of improvement through effort and strategy use. This mindset encourages them to view difficulties or challenges as part of the learning process, to persevere in the face of challenges, and to display adaptive learning behaviors that promote academic access (Blackwell et al., 2007; Yeager & Dweck, 2020). Overall, empirical research has shown that a fixed mindset is associated with less effort and poor academic performance, whereas a growth mind is associated with greater effort and improved academic performance (Blackwell et al., 2007; Sisk et al., 2018; Yeager et al., 2019).

Extant literature suggests that students’ mindsets may function as mediators in the association between autonomy support and academic achievement. Teacher and parent autonomy support promote students’ sense of agency and motivation (Ryan & Deci, 2020; Vansteenkiste et al., 2012), which, in turn, contributes to adaptive learning beliefs. Autonomy-supportive behaviors—such as validating students’ perspectives and encouraging self-initiation—help students feel that their actions are internally motivated rather than externally imposed. When students experience this form of support, they are more inclined to interpret challenges as opportunities for growth instead of threats to their ability. This promotes the development of a growth mindset—the belief that the ability and intelligence can improve with effort and learning, which has been linked to greater persistence and academic success (Dweck & Yeager, 2019; Yeager et al., 2019). Simultaneously, autonomy support may weaken fixed mindset belief (Zarrinabadi et al., 2021), by reducing external pressure and fear of failure. As students feel less judged, they become less inclined to interpret setbacks as reflections of low innate ability. This shift enhances resilience and sustained effort, ultimately promoting academic performance.

Students’ effort may mediate the association between autonomy support and student achievement. Grounded in self-determination theory, Grolnick and Slowiaczek (1994) proposed two models to explain the effects of parent involvement: a direct effect model and an indirect effect model. The direct model postulates that parent involvement influences academic outcomes by explicitly teaching academic skills, such as offering homework assistance. The indirect model postulates that parent involvement shapes outcomes by promoting students’ motivation—through autonomy support and encouragement to invest effort in academic tasks (e.g., homework). Synthesizing evidence across studies, Raftery et al. (2012) reported that “parent involvement may likely have its largest effects by facilitating the attitudes and values children need to put forth effort in school” (p. 348). Likewise, teacher autonomy support may promote student achievement indirectly by fostering greater academic effort. Autonomy-supportive teaching—by validating students’ perspectives, encouraging self-initiated learning, and minimizing external pressures—has been found to promote students’ intrinsic motivation and engagement (Ryan & Deci, 2020). This sense of ownership enhances students’ willingness to invest effort in academic tasks, which in turn predicts improved academic performance.

#### 1.1.3. Integrating Self-Determination and Mindset Theories

Whereas self-determination theory and mindset theory are frequently treated as separate traditions, together they offer a coherent account of how autonomy-supportive environments can influence students’ beliefs and behaviors in ways that ultimately promote achievement. From a self-determination perspective, autonomy support from teachers and parents creates a need-supportive context by acknowledging students’ perspectives and encouraging self-initiation. Such practices are theorized to satisfy students basic psychological needs—particularly autonomy—and to foster adaptive motivational and engagement (Ryan & Deci, 2020; Vansteenkiste et al., 2012). Importantly, these need-supportive experiences can also influence how students interpret difficulty: when students feel respected and agentic, academic challenges and difficulties are less likely to be experienced as threats and more likely to be viewed as opportunities to learn and improve.

Mindset theory complements this account by specifying how the need-supportive environment translates into achievement-relevant behavior through students’ beliefs about ability. Students with stronger growth mindset beliefs (i.e., ability can improve through effort and learning) are more inclined to interpret difficulties as informative and surmountable, which promotes persistence and sustained effort. In contrast, students with stronger fixed mindset beliefs tend to view difficulties as evidence of low ability, which can undermine effort and increase disengagement (Dweck & Yeager, 2019; Yeager & Dweck, 2020). Hence, autonomy-supportive practices may foster more adaptive mindsets by creating a psychological climate that reduces evaluative threat and supports competence development. These mindset beliefs, in turn, help explain whether students respond to challenges with greater effort versus avoidance or withdrawal (Blackwell et al., 2007; Yeager et al., 2019).

Together, these two perspectives jointly guide our framework: teacher and parent autonomy support are expected to provide a need-supportive context that promotes more adaptive homework mindsets (higher growth mindset, lower fixed mindset), which then fosters greater homework effort and higher achievement. This integrative approach clarifies both the contextual antecedent (autonomy-supportive socialization) and the intrapersonal mechanism (ability beliefs) through which students translate homework challenges into sustained effort and performance outcomes.

### 1.2. Studies Pertaining to Homework

While no prior study has directly tested whether homework mindsets and homework mediate the link between autonomy support and academic achievement, existing work implies these connections. Several studies have identified a positive relationship linking teacher autonomy support in homework, homework effort, and academic achievement. For example, Hagger et al. (2015), in a study of 220 Pakistani high school students, reported that teacher autonomy support enhanced autonomous motivation for mathematics and homework, which in related to higher grades. Similarly, Xu (2016) reported positive associations between teacher autonomy support, homework effort, and mathematics achievement among 918 Chinese middle school.

Parallel evidence exists for parents. In a study of 160 U.S. students in Grade 6, Grolnick et al. (2014) linked parent autonomy support to students’ engagement and academic achievement. Xu et al.’s (2024) meta-analysis likewise showed a positive association between parent autonomy support and achievement. In addition, Feng et al. (2019) reported that among 666 Chinese middle school students, parent autonomy support was positively associated with homework effort.

Importantly, involving 3018 Chinese middle school students, Xu and Corno (2022) examined the roles of teacher and parent autonomy support along with homework effort. Their results indicated that mathematics achievement was positively linked to homework effort, but not directly to teacher or parent autonomy support—suggesting that the autonomy support—achievement link may be reflected primarily in indirect associations via homework effort.

At the same time, research on homework mindsets—though limited—suggests that they may shape students’ effort and achievement (Bempechat, 2019). Luo et al. (2014), involving 2648 Singaporean secondary students, found that a fixed mindset related to more homework distraction and lower achievement, while a growth mindset related to greater effort and, in turn, higher achievement. Park et al. (2023), in a U.S. sample of 2176 elementary and secondary students, further showed that intrusive parent homework support was more detrimental to academic achievement over time for those with a fixed mindset. Whereas their focus was on intrusive rather than autonomy-supportive involvement, the findings point to the potential mediating roles of homework mindsets in the broader association between parental involvement and achievement, with autonomy support emerging as a particularly important form of involvement in this pathway.

Together, these results point to plausible pathways linking autonomy support to mindsets and effort, and ultimately to achievement. However, the joint roles of teacher and parent autonomy support, homework mindsets, and homework effort have not been investigated within a single integrative model—a gap the current study addresses.

### 1.3. The Current Study

**Figure 1:**
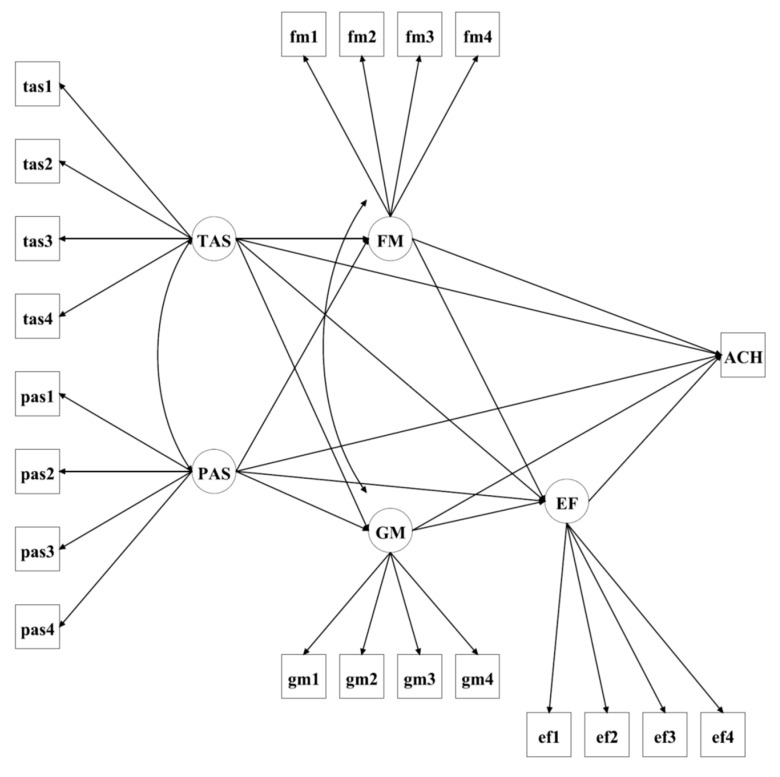
Structural model of the relationships among teacher autonomy support (TAS), parent autonomy support (PAS), fixed mindset (FM), growth mindset (GM), effort (EF), and achievement (ACH).

To extend existing research and fill this gap, the current study examines these associations within an integrated model, focusing on the mediating roles of homework mindsets and effort in the link between autonomy support and student achievement (Figure 1). Informed by the aforementioned theoretical and empirical insights, we hypothesized that teacher and parent autonomy support would be inversely associated with fixed mindset and positively associated with growth mindset, homework effort, and academic achievement. We further hypothesized that homework mindsets and effort would mediate the effects of autonomy support on student achievement. Specifically, teacher and parent autonomy support were hypothesized to be positively related to growth mindset and directly to homework effort, both of which would be positively related to student achievement. Additionally, autonomy support from teachers and parents was expected to be negatively related to fixed mindset, which would, in turn, be negatively related to homework effort and ultimately achievement. Finally, we hypothesized homework mindsets to mediate the link between autonomy support and homework effort.

## 2. Method

### 2.1. Participants and Procedure

The current investigation involved 988 students (47.6% female; all Han nationality) from 25 classes in southwestern China, distributed across Grade 7 (33.7%), Grade 8 (39.6%), and Grade 9 (26.7%). The mean age was 13.7 years (SD = 0.9). On average, fathers or male guardians had 15.8 years of education (SD = 2.6), whereas mothers or female guardians had an average of 15.6 years (SD = 2.6).

In line with prevailing practices in Chinese secondary education, the classes involved in this study promoted homework completion tasks in a structured setting and emphasized timely submission. Parents or guardians were advised to facilitate this by establishing a dedicated homework space and by supplying relevant educational materials. With regard to mathematics homework, nearly all students (96.4%) reported receiving assignments at least five days weekly and spending an average of 43 min daily (SD = 26). These patterns mirror results from earlier research in China (Yang & Tu, 2020).

After securing approval from the institutional review board (MYRG2017-00122-FED), informed consent was obtained from parents or guardians before data collection. Trained research assistants administered the survey measures (described in the next session) during normal class periods. To minimize potential bias, teachers were asked to leave the room during administration. Student responses were linked to final mathematics grades using participant identification codes. The final participation rate was 93%.

### 2.2. Measures

#### 2.2.1. Teacher Autonomy Support

Teacher autonomy support was measured using a four-item scale (Table 1; Xu, 2016) that captured students’ perceptions of autonomy support regarding mathematics assignments (e.g., teachers attending to students’ views in approaching mathematics homework and promoting their initiative; α/ω = 0.95/0.95). The reliability coefficients aligned with prior findings among Chinese students (0.83 ≤ α/ω ≤ 0.85; Xu, 2016; Xu & Corno, 2022).

#### 2.2.2. Parent Autonomy Support

Parent autonomy support was measured using a four-item scale (Xu et al., 2017), designed to capture students’ perceptions of autonomy-supportive behaviors from parents while working on mathematics assignments (e.g., validating their perspectives and promoting personal initiative; α/ω = 0.94/0.94). The reliability coefficients aligned with prior research among Chinese students (α/ω = 0.91; Xu & Corno, 2022; Xu et al., 2017).

#### 2.2.3. Fixed and Growth Homework Mindsets

Drawing on extant research on mindset theory (De Castella & Byrne, 2015; Dweck, 1999; Luo et al., 2014), the scale incorporated two subscales assessing fixed and growth mindsets. The fixed mindset subscale captured the belief that one’s ability to complete mathematics homework is innate and cannot be changed (four-item; α/ω = 0.93/0.93). In contrast, the growth mindset subscale reflected the belief that this ability can be developed through effort and learning (four-item; α/ω = 0.94/0.95). Confirmatory factor analysis provided robust support for the distinctiveness of these two constructs (CFI = 0.983; RMSEA = 0.046; SRMR = 0.030).

#### 2.2.4. Homework Effort

Informed by and built upon previous research (Trautwein et al., 2006; Xu, 2024), four items measured the extent to which students seriously worked on mathematics homework (e.g., with respect to student initiative to complete assignments; α/ω = 0.88/0.88). The reliability estimates aligned with prior studies among Chinese students (0.81 ≤ α/ω ≤ 0.86; Xu, 2024, 2025; Xu & Corno, 2022).

#### 2.2.5. Mathematics Achievement

Final mathematics grades were extracted from official school records maintained by teachers. Grades were reported on a standardized 5-point scale, with 1 indicating an F and 5 indicating an A. Although students were sampled from different classes, the grades were considered comparable due to the consistent use of standardized curriculum and grading policies commonly applied throughout Chinese schools.

### 2.3. Data Analysis

We carried out data analyses applying structural equation modeling with the Bayesian estimator in Mplus 8.11. This approach was selected for two main reasons. First, Bayesian estimation offers greater robustness (e.g., more accurate uncertainty quantification) than traditional maximum likelihood-based estimators when the number of clusters is relatively small—as in the current investigation, which included 25 classes. Under such conditions, standard corrections for clustering (e.g., the COMPLEX option with MLR) may result in biased standard errors and test statistics due to the limited number of level-2 units (Asparouhov & Muthén, 2023). Furthermore, Bayesian methods are especially suited for estimating complex models (e.g., multiple mediated paths and latent variables), allowing for the incorporation of prior distributions and generating full posterior distributions for parameters.

As illustrated in Figure 1, the structural model was specified with teacher autonomy support and parent support as exogenous variables; fixed mindset, growth mindset, and homework effort as mediators; and mathematics achievement as the outcome variable. Model fit was assessed by applying Bayesian and conventional indices (CFI ≥ 0.95, TLI ≥ 0.95, RMSEA ≤ 0.06; Hu & Bentler, 1999). Posterior predictive checking was performed by comparing observed and replicated chi-square values. Path coefficients were interpreted based on posterior means and their 95% credibility intervals (Crl). A 95% Crl indicates that, given the data and the specified model (and prior), there is a 95% posterior probability that the parameter falls within this range. Indirect effects were examined within Bayesian framework, with evidence for an effect inferred when the 95% Crl excluded zero. Less than 1% of data were missing, and these were addressed through Bayesian multiple imputation.

## 3. Results

As displayed in Table 2, significant correlations emerged among all study variables, including autonomy support (from both teachers and parents), mindsets, homework effort, and mathematics achievement, except for a non-significant relationship between fixed and growth mindsets. Specifically, teacher autonomy support was positively associated with mathematics achievement (*r* = 0.23, *p* < 0.01), homework effort (*r* = 0.38, *p* < 0.01), and growth mindset (*r* = 0.30, *p* < 0.01), whereas negatively associated with fixed mindset (*r* = −0.18, *p* < 0.01). Likewise, parent autonomy support showed positive associations with mathematics achievement (*r* = 0.25, *p* < 0.01), homework effort (*r* = 0.32, *p* < 0.01), and growth mindset (*r* = 0.32, *p* < 0.01), and a negative association with fixed mindset (*r* = −0.20, *p* < 0.01). Additionally, fixed mindset was negatively related to mathematics achievement (*r* = −0.21, *p* < 0.01) and homework effort (*r* = −0.13, *p* < 0.01), while growth mindset showed positive associations with both mathematics achievement (*r* = 0.24, *p* < 0.01) and homework effort (*r* = 0.20, *p* < 0.01). Moreover, homework effort was positively associated with mathematics achievement (*r* = 0.19, *p* < 0.01).

Guided by our model (see Figure 1) and the zero-order correlation results, we carried out a structural model analysis using Bayesian estimation. In this model, teacher autonomy support and parent autonomy support were specified as exogenous variables; fixed mindset, growth mindset, and homework effort functioned as mediators; and mathematics achievement was the outcome variable. Model fit was assessed by applying both posterior predictive checking and conventional fit indices provided under Bayesian estimation. Whereas the posterior predictive *p*-value was 0.000 (95% CI for the difference between observed and replicated chi-square = [391.711, 417.646], conventional fit indices indicated good model fit: CFI = 0.978 (90% CI [0.977, 0.979], TLI = 0.974 (90% CI [0.972, 0.975], and RMSEA = 0.046 (90% CI [0.045, 0.047]).

**Figure 2:**
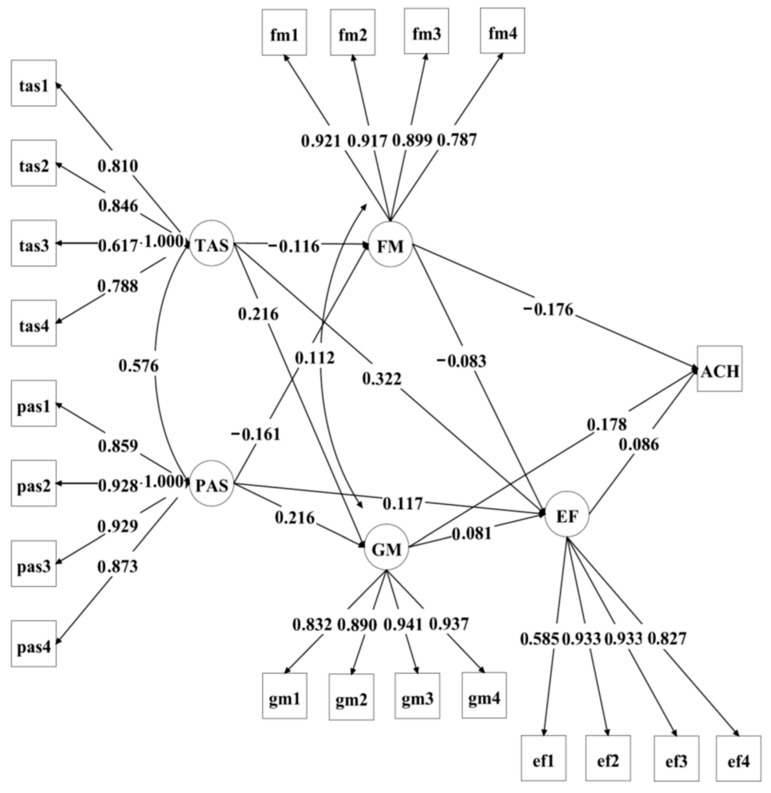
Standardized estimates for statistically significant structural paths for teacher autonomy support (TAS), parent autonomy support (PAS), fixed mindset (FM), growth mindset (GM), effort (EF), and achievement (ACH).

The findings of the mediation analysis were displayed in Figure 2 and Table 3. As seen from Figure 2, both teacher autonomy support and parent autonomy support were significantly associated with students’ fixed mindset, growth mindset, and homework effort. In turn, both mindsets and homework effort were significantly related to mathematics achievement. In addition, fixed and growth mindset were significantly associated with homework effort, and homework effort was significantly related to mathematics achievement.

Table 3 includes the standardized estimates, posterior standard deviations, Bayesian one-tailed *p*-values, and 95% credibility intervals (Crls) for all direct, indirect, and total effects. Notably, teacher autonomy support and parent autonomy support showed statistically credible total effects on mathematics achievement, as indicated by 95% Crls that excluded zero (TAS → ACH: Estimate = 0.174, Crl = [0.093, 0.253]; PAS → ACH: Estimate = 0.149, Crl = [0.068, 0.225]). These total effects were largely driven by multiple significant indirect paths involving fixed mindset, growth mindset, and homework effort. Asterisks in Table 3 indicate that the 95% Crl for a given path estimate excludes zero, signifying statistical credibility under Bayesian estimation.

Whereas the direct effects of teacher autonomy support (Estimate = 0.085, Crl = [0.000, 0.169] and parent autonomy support (Estimate = 0.069, Crl = [−0.011, 0.145]) on mathematics achievement did not reach statistical significance at the 95% credibility interval level, their indirect effects were consistently robust. Specifically, both teacher autonomy support and parent autonomy support exhibited significant indirect effects on mathematics achievement via fixed mindset (TAS: Estimate = 0.020, Crl = [0.005, 0.038]; PAS: Estimate = 0.028, Crl = [0.012, 0.047]) and growth mindset (TAS: Estimate = 0.038, Crl = [0.020, 0.060]; PAS: Estimate = 0.038, Crl = [0.020, 0.060]). Additional significant mediation pathways included homework effort (e.g., TAS → EF → ACH, Estimate = 0.027, Crl = [0.006, 0.052]) and more complex, sequential mediations involving both homework mindsets and effort (e.g., TAS → GM → EF → ACH, Estimate = 0.001, Crl = [0.000, 0.004]). These results highlight the important role of students’ homework mindsets and effort in linking autonomy support to academic achievement.

Teacher autonomy support and parent autonomy support were significantly related to homework effort through both direct and indirect pathways. Teacher autonomy support showed a significant effect on homework effort (Estimate = 0.322, 95% Crl [0.243, 0.400]) as well as a significant total indirect effect (Estimate = 0.027, 95% Crl [0.009, 0.047]). To be specific, teacher autonomy support was indirectly associated with homework effort through fixed mindset (Estimate = 0.009) and growth mindset (Estimate = 0.017). Likewise, parent autonomy support had a significant effect on homework effort (Estimate = 0.117, 95% Crl [0.038, 0.195]) and a significant total indirect effect (Estimate = 0.030, 95% Crl [0.011, 0.053]). These indirect effects occurred via both fixed mindset (Estimate = 0.013) and growth mindset (Estimate = 0.017). These results suggest that both forms of autonomy support significantly promote students’ homework effort, both directly and indirectly through their effects on students’ fixed and growth mindsets.

## 4. Discussion

### 4.1. Interpretation

As hypothesized, both teacher and parent autonomy support were positively related to mathematics grades, primarily through indirect paths involving homework mindsets and effort. Notably, autonomy support was linked to greater endorsement of a growth mindset and increased homework effort, which in turn led to higher grades. These results support existing evidence that autonomy-supportive environments nurture adaptive motivational beliefs and sustained effort (Feng et al., 2019; Luo et al., 2014; Yeager et al., 2019), which are known predictors of academic success (Luo et al., 2014; Rosário et al., 2018; Xu & Corno, 2022). Importantly, this current study bridges a gap in the literature by simultaneously examining whether both fixed and growth mindsets, along with homework effort, mediate the link between the effects of teacher and parent autonomy support on student achievement within a single, integrated model.

Beyond establishing significant indirect associations, our results reinforce the integrative framework presented in the Introduction. From a self-determination theory (SDT) perspective, autonomy support operates as a distal contextual input that facilitates adaptive motivation and engagement rather than through a direct, unmediated link with achievement. Mindset theory complements this perspective by highlighting a cognitive-motivational mechanism (interpretation of difficulty/ability): autonomy supportive environments may foster more malleable views of ability, which buffer students against setbacks and support persistence during challenging homework. Overall, this pattern aligns with a joint SDT × mindset pathway linking need-supportive environments to achievement through students’ interpretations of difficulty and consequent effortful engagement.

Our findings revealed that autonomy support was negatively associated with fixed mindset, and that fixed mindset was negatively associated with homework effort and mathematics grades, supporting the hypothesized detrimental role of endorsing fixed ability beliefs. Importantly, homework mindsets functioned as mediators in the association between autonomy support and homework effort, indicating that fostering growth-oriented beliefs may be a central mechanism through which autonomy support promotes student effort and performance. This pattern suggests that autonomy support may be more consequential when it reshapes how students interpret homework difficulty, reframing it from a threat to ability to an opportunity for learning improvement. A plausible explanation is that autonomy support in homework encourages students to perceive homework as self-directed, effort-based, and an opportunity for learning and growth rather than a fixed measure of ability. This explanation is supported by existing research that autonomy support fosters intrinsic motivation (Ryan & Deci, 2020) and promotes growth-oriented beliefs (Kim et al., 2017; Zarrinabadi et al., 2021). These beliefs help students reinterpret academic effort as meaningful investment in learning rather than a sign of inadequacy or low ability (Brooks et al., 2012; Yeager & Dweck, 2020). Consequently, students are more inclined to put forth sustained homework effort, leading to better academic achievement.

Among indirect effects, growth-mindset pathways were generally the largest, with fixed-mindset pathways close behind in some cases, suggesting that growth-oriented beliefs may be especially relevant for homework effort and mathematics achievement. In terms of magnitude, the most practically notable effects involved autonomy support in relation to homework effort. Specifically, teacher autonomy support showed a medium-sized total association with homework effort (d = 0.57), including medium-sized direct association (d = 0.50), while parent autonomy support showed smaller associations (total d = 0.24; direct d = 0.19). In contrast, associations with mathematics grades were small in magnitude but consistently nontrivial, with total effect of d = 0.27 (teacher) and d = 0.24 (parent). Importantly, these total associations with mathematics grades were accounted for largely by small-to-approaching-moderate indirect effects (total indirect d = 0.31 for teacher; d = 0.34 for parent), particularly pathways involving growth mindset (d = 0.24 for both teacher → growth mindset → achievement and parent → growth mindset → achievement) and, to a somewhat lesser extent, fixed mindset (d = 0.14–0.24). Indirect effects operating through homework effort alone were smaller (e.g., teacher → effort → achievement, d = 0.14; parent → effort → achievement; d = 0.10), and the serial paths via mindset → effort → achievement were very small (d ≈ 0.06). Taken together, these patterns suggest that primary practical leverage lies in the associations between autonomy support and students’ mindsets and overall homework effort, rather than in longer chain pathways. Although the achievement effects were small, they may still hold practical significance in educational settings, where modest differences in motivation and engagement can accumulate over time and across students (Götz et al., 2022; Xu & Corno, 2022).

Notably, teacher autonomy support showed somewhat stronger associations than parent autonomy support, highlighting the potential salient role of teachers in relation to students’ motivational, behavioral, and achievement outcomes. One likely explanation is that, by the virtue of their subject expertise, teachers occupy a central position in shaping students’ academic development, especially in challenging disciplines like mathematics (Davidovitch & Yavich, 2017; Xu, 2025). When teachers promote autonomy by encouraging student self-direction and fostering a feeling of control over mathematics homework, students become more inclined to believe in their ability to improve through sustained effort (Cheon et al., 2020; Xu, 2016). Consequently, they are more likely to invest greater effort in following through their homework assignments.

A complementary explanation is that autonomy support may be more effective when accompanied by optimal structure—clear expectations, guidance, and informational feedback—so that students experience support for both autonomy and competence (Jang et al., 2010). Teachers are often able to integrate autonomy support with task-relevant structure in mathematics (e.g., feedback aligned with learning goals), while parent autonomy support may be less closely tied to the specific instructional demands. Accordingly, the stronger teacher pathways may reflect not only who provides support, but also the extent to which autonomy support is embedded within competence-supporting structure.

In contrast, parent involvement in homework typically takes place under different constraints than teacher involvement. Because it occurs outside the instructional setting, it is frequently shaped by immediate needs (e.g., when a student is stuck) by parents’ time, confidence, and subject-matter familiarity (Jay et al., 2018; Syla, 2023; Xu, 2025). Accordingly, parental support may more often involve encouragement, reminders, or monitoring—approaches that can promote positive attitudes toward homework but may less consistently translate into greater effort on demanding mathematics assignments. These constraints may become more pronounced in middle school as mathematics homework increases in difficulty and parents’ perceived ability to help declines (Hill & Tyson, 2009).

Another possible explanation lies in the potential role of Confucian values, which emphasize deference to teachers as the authoritative sources of knowledge and guidance (Y. Wang et al., 2024; Xu, 2025). Within this cultural context, students may be more attuned to teachers’ homework guidance, particularly in challenging disciplines such as mathematics, viewing it as more salient and relevant to their homework success. As such, autonomy support from teachers may carry greater motivational weight, be more readily internalized. and more effectively promote homework effort.

In many Western contexts, particularly in mainstream middle-class settings where individualism, self-reliance, personal initiative are emphasized, autonomy support tends to be viewed as a normative and desirable feature of both schooling and parenting (Chirkov & Ryan, 2001; Reeve et al., 2014). Accordingly, parent autonomy support may be particularly salient for homework-related initiative in these contexts than in Confucian-heritage settings, where teachers are traditionally accorded stronger academic authority. Thus, the relative contributions of teacher versus parent autonomy support to homework effort may differ from those observed in Confucian-heritage samples, potentially with a stronger role for parent autonomy support. Future cross-cultural replications are needed to examine cultural moderation of these pathways.

Whereas the direct paths from teacher and parent autonomy support to mathematics grades were not statistically significant, their total associations with mathematics grades were significant, largely driven by robust indirect pathways through homework mindsets and effort. This pattern suggests that autonomy support is more closely linked to achievement through its associations with students’ beliefs and homework-related behaviors, rather through a direct relation with achievement. In other words, the links between teacher and parent autonomy support and mathematics grades appear to operate primarily through indirect pathways via homework mindsets and homework effort. One possible explanation is that students may interpret homework autonomy support differently based on their preexisting learning beliefs. Some may experience it as a sign of trust that strengthens ownership and responsibility for homework. Others, particularly those accustomed to directive teaching, may perceive the same practices as insufficient structure or guidance. This response may be more common in China, where instruction is often highly structured and teacher-centered and is shaped by cultural norms emphasizing authority and compliance (Chan, 2019; D. Wang, 2011). As such, autonomy support may only become effective when it helps reshape students’ views of learning and their roles in it, especially their beliefs about whether academic ability can improve with effort in cognitively demanding subjects like mathematics.

Theoretically, this pattern is particularly informative. Had autonomy support shown a robust direct association with grades, it could have pointed to additional unmeasured pathways, such as instructional practices more directly tied to performance evaluation, grading-related processes, or other classroom factors not represented in the current model. Instead, the predominance of indirect pathways through homework mindsets and effort suggests that autonomy support relates to achievement primarily by shaping students’ psychological interpretations and homework engagement, consistent with both SDT and mindset theory.

Importantly, the nonsignificant direct effect is theoretically meaningful rather than disappointing. From SDT standpoint, autonomy support is not expected to enhance achievement in a direct, unmediated way; rather, it operates as a distal contextual input whose influence is expressed through students’ need-relevant appraisal and subsequent engagement. This does not imply that autonomy support is unimportant; instead, it suggests that its effects are transmitted through students’ beliefs and effort. Likewise, mindset theory proposes that messages from important others influence students’ beliefs about ability and the meaning of difficulty—beliefs that can relate to achievement both directly and indirectly by promoting (or undermining) persistence and effort. Taken together, the mediated pattern observed here provides particularly strong evidence for the SDT × mindset assumption that supportive social environments “gets under the skin” by altering internal psychological processes—students’ beliefs and effort engagement—rather than exerting a direct effect on grades.

As shown in Figure 2, the multiple mediational paths through homework mindsets and effort underscore the importance of targeting these psychological constructs in homework research and practice aimed at improving student achievement. The significant, albert smaller, paths involving fixed mindset and homework effort further emphasize the multifaceted nature of motivational and behavioral mechanisms in homework contexts.

Overall, by combining self-determination and mindset theories, our study offers a deeper understanding of how teacher and parent autonomy support are indirectly associated with mathematics grades through homework mindsets and effort. To our knowledge, this is the first to simultaneously test the joint statistical mediating roles of both growth and mixed mindsets, together with homework effort, in linking autonomy support from both teachers and parents to academic achievement within a single empirical model. As discussed above, this integrated framework highlights the interconnected nature of autonomy support, homework mindsets, and effort, with growth mindset emerging as a particularly salient pathway in the homework context.

### 4.2. Practical Implications

Because this is the first investigation to examine how homework mindsets and effort mediate the autonomy support–achievement link, further empirical work is needed to validate and extend these findings. Still, our findings may offer useful guidance for homework practices.

First, our findings suggest that improving achievement may require targeting the processes most proximal to performance—students’ homework mindsets and homework effort. For teachers, autonomy support can be exacted through providing clear rationales, encouraging self-initiated learning, and framing mistakes as a normal part of learning (Feng et al., 2019; Xu et al., 2021). For parents, autonomy-supportive involvement may include helping students set learning goals for homework and establishing routines that provide structure while allowing for flexibility, independence, and self-direction (Froiland, 2011). Together, these practices may strengthen students’ ownership and sense of control over their homework while fostering growth mindset and sustained effort—key pathways to achievement identified in the current investigation. Such strategies may be especially important in demanding subjects such as mathematics, where persistence and follow-through are essential for success.

Second, our findings underscore the importance of promoting a growth mindset in homework contexts. Teachers and parents can support this by reinforcing the belief that academic ability is malleable and can improve with effort and effective strategies. By encouraging students to view homework challenges or setbacks as opportunities for growth, as well as by emphasizing their effort over innate ability, they can help cultivate a mindset that promotes sustained homework engagement and academic resilience.

Third, the significant role of homework effort in linking autonomy support to achievement underscores the importance of nurturing students’ motivation and responsibility. Rather than emphasizing compliance, teachers and parents can promote effort by supporting autonomy. Given the stronger relationship between teacher autonomy support and homework effort found in this study, it is particularly important for teachers to create classroom environments that empower students to take ownership of learning (e.g., valuing their preferences and ideas, offering encouragement, and fostering self-direction).

Fourth, whereas teacher autonomy support seemed more influential than parent autonomy support in the current investigation, particularly within the Confucian cultural context, it remains important for parents to offer support that is respectful of students’ autonomy. Coordinated efforts between school and home that emphasize encouragement and student agency are likely to be more effective in reinforcing students’ adaptive motivational beliefs, sustained effort, and academic growth.

Finally, it is crucial to create a space in which students feel comfortable discussing their learning experiences (Kackar et al., 2011; Mammadov & Schroeder, 2023), especially their perceptions of autonomy support from teachers and parents and how these perceptions shape their learning beliefs and effort. Encouraging student voice helps teachers and parents better understand the motivational dynamics that drive homework effort and performance, allowing them to better tailor their support to students’ needs. Open dialogue not only affirms students’ perspectives but also helps uncover potential disconnects between well-intentioned adult support and student interpretations. When students feel genuinely heard, they become more inclined to experience autonomy support as authentic, which in turn promotes homework effort and performance. Active listing to student voice, along with autonomy support that is responsive and empathetic, can be instrumental in promoting homework effectiveness and long-term academic success.

### 4.3. Limitations and Future Research

Despite its contributions, the current study has several limitations that merit consideration. First, autonomy support, mindsets, and homework effort were measured through student self-reports. Whereas self-reports are a standard method in studies on autonomy support (Martínez-López et al., 2023; Xu et al., 2021), mindsets (De Castella & Byrne, 2015; Dweck, 1999), and academic effort (Marsh et al., 2016; Trautwein et al., 2006), they are susceptible to shared method variance and systematic response tendencies (Marsh et al., 2016). While student achievement was obtained from an external source, subsequent studies could enhance validity by incorporating multiple data sources, like parent or teacher reports, or observational data when feasible. Yet, given that homework typically takes place outside the direction supervision of adults, such alternatives may be challenging to implement in homework-related studies.

Second, because all variables (except mathematics achievement) were assessed at a single time point, the mediation analyses cannot establish temporal precedence or capture a process unfolding over time, alternative directional or reciprocal models remain plausible. Hence, the estimated indirect effects should be interpreted as conditional, theory-consistent associations (i.e., statistical mediation), rather than as evidence of causal mediation or an underlying causal mechanism. Experimental or longitudinal or designs are needed to disentangle the temporal ordering of these variables and to better capture the developmental nature of these processes over time. For instance, further investigations could explore whether increases in autonomy support lead to corresponding growth in students’ mindsets, sustained effort, and improved academic performance throughout the school years.

Third, the clustered data structure suggests that class-level factors may contribute to the observed associations. With only 25 classes, however, estimating a full multilevel SEM with multiple latent mediators would be underpowered and potentially unstable; future studies with a large number of classes should apply multiple modeling to examine whether the proposed pathways replicate at both the student and the classroom levels.

Finally, this study was carried out among students enrolled in Chinese middle schools. Rooted in a Confucian tradition that positions teachers as central academic authorities, the patterns observed in this sample may differ in countries with different views on the roles of teachers, parents, and students. Cultural differences in the value placed on autonomy support, mindsets, effort, and academic success may shape the nature and strength of these associations (Ryan & Deci, 2020; Yeager & Dweck, 2020). Future cross-cultural research is needed to clarify whether the mediating mechanisms observed in this study are culturally specifical or hold across different educational systems.

## 5. Conclusions

Informed by self-determination and mindset theories, this study addressed a key gap by examining a theoretically specified model linking teacher and parent autonomy support to achievement through students’ homework mindsets and effort. Findings indicated positive indirect associations between both teacher autonomy support and parent autonomy support and mathematics grades, operating through fixed mindset, growth mindset, and homework effort. Moreover, autonomy support from both sources was associated with higher homework effort both directly and through its associations with fixed and growth mindsets.

## Figures and Tables

**Table 1:** Scales and Reliability Estimates.

Scales	Items	
Teacher autonomy support ^a^	tas1	“My math teacher encourages me to ask questions about HW assignments.”
	tas2	“My math teacher listens to my ideas about HW assignments.”
	tas3	“My math teacher listens to how I would like to do HW assignments.”
	tas4	“My math teacher conveys confidence in my ability to do with HW assignments.”
Parent autonomy support ^a^	pas1	“My parents encourage me to ask questions about math HW assignments.”
	pas2	“My parents listen to my ideas about math HW assignments.”
	pas3	“My parents listen to how I would like to do math HW assignments.”
	pas4	“My parents convey confidence in my ability to do with math HW assignments.”
Fixed mindset ^b^	fm1	“I don’t think I personally can do much to increase my ability to do math HW.”
	fm2	“My ability to do math HW is something about me that I personally can’t change very much.”
	fm3	“To be honest, I don’t think I can really change my ability to do math HW.”
	fm4	“I can learn new things, but I can’t really change my basic math ability.”
Growth mindset ^b^	gm1	“With enough time and effort I think I could significantly improve my ability to do math HW.”
	gm2	“I believe I can always substantially improve on my ability to do math HW.”
	gm3	“Regardless of my current ability to do math HW, I think I have the capacity to change it quite a bit.”
	gm4	“I believe I can change my basic math ability considerably over time.”
Effort ^a^	ef1	“In math HW, I invest much effort to understand everything.”
	ef2	“I’ve recently been doing my math HW to the best of my ability.”
	ef3	“I do my best on my math HW.”
	ef4	“I always try to finish my math HW.”

Note. HW = homework. ^a^ Rating: From 1 (strongly disagree) to 4 (strongly agree). ^b^ Rating: From 1 (not at all true of me) to 7 (very true of me).

**Table 2:** Descriptive Statistics and Correlations.

Variables	Mean	SD	1	2	3	4	5
1 Teacher autonomy support	3.20	0.72	−				
2 Parent autonomy support	3.23	0.89	0.52 **	−			
3 Fixed mindset	2.15	1.61	–0.18 **	–0.20 **	−		
4 Growth mindset	4.70	2.01	0.30 **	0.32 **	0.03	−	
5 Effort	3.38	0.66	0.38 **	0.32 **	–0.13 **	0.20 **	−
6 Achievement	4.53	0.89	0.23 **	0.25 **	–0.21 **	0.24 **	0.19 **

Note: N = 988. SD = Standard deviations. ** *p* < 0.01.

**Table 3:** Standardized Estimates and 95% Credibility Intervals for Direct, Indirect, and Total Effects.

Model Path	Estimate	Posterior S.D.	One-Tailed *p*-Value	95% Crl		Significance	Cohen’s d
Effects from TAS to EF							
Total	0.349	0.039	0.000	0.272	0.425	*	0.57
Total indirect	0.027	0.010	0.002	0.009	0.047	*	0.17
Indirect 1: TAS → FM → EF	0.009	0.005	0.008	0.001	0.022	*	0.11
Indirect 2: TAS → GM → EF	0.017	0.008	0.008	0.003	0.034	*	0.14
Direct: TAS → EF	0.322	0.041	0.000	0.243	0.400	*	0.50
Effects from PAS to EF							
Total	0.147	0.039	0.000	0.071	0.224	*	0.24
Total indirect	0.030	0.011	0.001	0.011	0.053	*	0.17
Indirect 1: PAS → FM → EF	0.013	0.007	0.004	0.003	0.028	*	0.12
Indirect 2: PAS → GM → EF	0.017	0.008	0.008	0.003	0.035	*	0.14
Direct: PAS → EF	0.117	0.040	0.003	0.038	0.195	*	0.19
Effects from TAS to ACH							
Total	0.174	0.041	0.000	0.093	0.253	*	0.27
Total indirect	0.088	0.018	0.000	0.056	0.125	*	0.31
Indirect 1: TAS → FM → ACH	0.020	0.009	0.004	0.005	0.038	*	0.14
Indirect 2: TAS → GM → ACH	0.038	0.010	0.000	0.020	0.060	*	0.24
Indirect 3: TAS → EF → ACH	0.027	0.012	0.007	0.006	0.052	*	0.14
Indirect 4: TAS → FM → EF → ACH	0.001	0.001	0.015	0.000 ^a^	0.002	*	0.06
Indirect 5: TAS → GM →EF → ACH	0.001	0.001	0.015	0.000 ^a^	0.004	*	0.06
Direct: TAS → ACH	0.085	0.043	0.025	0.000	0.169		0.13
Effects from PAS to ACH							
Total	0.149	0.040	0.000	0.068	0.225	*	0.24
Total indirect	0.079	0.015	0.000	0.052	0.109	*	0.34
Indirect 1: PAS → FM → ACH	0.028	0.009	0.000	0.012	0.047	*	0.20
Indirect 2: PAS → GM → ACH	0.038	0.010	0.000	0.020	0.060	*	0.24
Indirect 3: PAS → EF → ACH	0.009	0.006	0.010	0.001	0.023	*	0.10
Indirect 4: PAS → FM → EF → ACH	0.001	0.001	0.011	0.000 ^a^	0.003	*	0.06
Indirect 5: PAS → GM →EF → ACH	0.001	0.001	0.015	0.000 ^a^	0.004	*	0.06
Direct: PAS → ACH	0.069	0.040	0.046	−0.011	0.145		0.11

Note: Crl = Credibility interval based on Bayesian estimation. Asterisks indicate that the 95% Crl excludes 0. ^a^ The indirect effect, though small, was identified as significant (*) due to a credibility interval that likely excluded zero with greater estimation precision.

## Data Availability

Data will be available upon request.
